# QTL Mapping for Leaf Rust Resistance in a Wheat Recombinant Inbred Line Population of Liang66-S/Hengmai28

**DOI:** 10.3390/plants15172684

**Published:** 2026-08-31

**Authors:** Cunyao Bo, Chenyang Pang, Xianghai Meng, Ding Li, Minghui Zhao, Chuncai Shen, Qiang Li, Wenchen Qiao

**Affiliations:** 1 National Key Laboratory of Wheat Improvement, College of Agronomy, Shandong Agricultural University, Tai’an 271018, China; bocunyao1989@126.com (C.B.); pangcy118@foxmail.com (C.P.); 2 Dryland Farming Institute, Hebei Academy of Agriculture and Forestry Sciences, Hengshui 053000, China; mengxianghai5229@163.com (X.M.); nxld854@163.com (D.L.); mnzhaominghui@163.com (M.Z.); shencc_0419@126.com (C.S.); 3 Hebei Provincial Key Laboratory of Crops Drought Resistance Research, Hengshui 053000, China

**Keywords:** common wheat, kompetitive allele-specific PCR (KASP), leaf rust resistance, marker-assisted selection (MAS), quantitative trait locus (QTL)

## Abstract

Leaf rust (LR), caused by *Puccinia triticina*, is a major constraint to global wheat production. Identifying quantitative trait loci (QTLs), conferring adult-plant resistance (APR), and developing breeder-friendly markers are essential for durable disease control. In this study, a recombinant inbred line (RIL) population derived from Liang66-S × Hengmai28 was evaluated for leaf rust severity across four environments. Maximum disease severity (MDS) showed continuous variation and moderate to high correlations among environments, indicating polygenic inheritance. Five APR QTLs were mapped on chromosomes 2BL, 3BS, 3BL, 7BL and 7DL, explaining 4.5–9.8% of the phenotypic variance (PVE) with LOD scores of 2.9–7.9. Among these, *QLr.DFI-2BL*, *QLr.DFI-3BS*, and *QLr.DFI-7BL* correspond to previously reported APR loci, whereas *QLr.DFI-3BL* and *QLr.DFI-7DL* represent potentially novel loci. *QLr.DFI-3BL* was detected across all four environments, while *QLr.DFI-3BS* and *QLr.DFI-7BL* showed the highest R^2^ (7.7–8.5% and 8.4–9.8%). Multiple defense-related genes within the QTL intervals for LR were identified, including the putative NBS-LRR, the putative BTB/POZ-MATH, F-box, receptor-like kinase, polyphenol oxidase, calcium-dependent protein kinase, peroxidase, and ethylene-responsive transcription factors. Based on the flanking markers, five KASP markers were developed. *K-LR-3BS* (for *QLr.DFI-3BS*) and *K-LR-7BL* (for *QLr.DFI-7BL*) successfully genotyped a natural population with 120 accessions, with call rates of 100% and 94.2%, and showed significant association with reduced leaf rust severity. However, further validation in diverse genetic backgrounds and populations is required before they can be considered for routine marker-assisted selection. These results provide two KASP markers as promising markers that require further validation in diverse backgrounds and highlight *QLr.DFI-3BS* and *QLr.DFI-7BL* as promising targets for fine mapping and eventual cloning of APR genes in wheat.

## 1. Introduction

Leaf rust, caused by the fungal pathogen *Puccinia triticina* (Pt), is a devastating disease characterized by wide distribution, high frequency, and sudden outbreaks, posing a serious threat to wheat production. Leaf rust occurs in nearly all wheat-growing regions worldwide, with particularly severe impacts in warm and humid environments such as the southeastern United States, the Pampas of South America, the Indo-Gangetic Plains of South Asia, and the North China Plain. The pathogen can cause yield losses exceeding 50% under severe epidemics [1]. More critically, global warming has intensified the epidemic of leaf rust in major wheat-growing regions worldwide, including the southern United States, South America, Canada, Eastern Europe, Egypt, and the entirety of China [2]. Rising temperatures and altered precipitation patterns have extended the seasonal window conducive to pathogen reproduction, enhanced overwintering survival of urediniospores, and accelerated disease cycles, leading to earlier onset and more frequent severe outbreaks in many temperate zones. Severe yield losses due to leaf rust outbreaks occurred globally in 2008, 2009, 2012, 2015, and 2023 [3]. Therefore, effective control of leaf rust is essential for safeguarding global food security.

Breeding resistant varieties is the most economical and effective strategy for managing leaf rust [4]. Wheat leaf rust resistance is primarily categorized into all-stage resistance (ASR) and adult-plant resistance (APR). ASR is typically governed by one or a few major genes and confers race-specific resistance based on the gene-for-gene theory, often resulting in high resistance or immunity to specific pathogen races [5]. This type of resistance imposes strong selection pressure on the pathogen and can rapidly lose effectiveness upon the emergence of new virulent races. For instance, the widely deployed genes *Lr1*, *Lr13*, and *Lr26* have successively become ineffective in many regions [6]. APR is characterized by susceptibility at the seedling stage but resistance at the adult stage and is controlled by multiple minor genes acting in concert [7]. Typically race-nonspecific, APR reduces disease severity through a prolonged latent period, reduced infection frequency, and decreased spore production, thus imposing low selection pressure on the pathogen and conferring durable and stable resistance [8]. Although individual APR genes have limited effects, pyramiding four to five such genes can achieve high to near-immune resistance levels. Since the early 2000s, the International Maize and Wheat Improvement Center (CIMMYT) has successfully developed high-yielding wheat materials with durable, high-level APR through gene pyramiding [9].

Resistance breeding is severely constrained by the narrow genetic basis and the limited availability of effective resistance sources. Since the initiation of genetic research on wheat leaf rust resistance in 1926, a total of 83 leaf rust resistance genes have been officially named internationally, most of which are ASR genes [10]. The well-characterized APR genes include *Lr12*, *Lr13*, *Lr22a*, *Lr22b*, *Lr34*, *Lr35*, *Lr37*, *Lr46*, *Lr48*, *Lr49*, *Lr67*, and *Lr68* [9,11,12]. Over ten Lr genes have been cloned, with most ASR genes encoding NLR proteins (e.g., *Lr1*, *Lr10*, *Lr21*), while APR genes encode diverse protein types [13,14]. Notably, *Lr34* (ABC transporter) and *Lr67* (hexose transporter) exhibit pleiotropic effects, conferring broad-spectrum and durable resistance to multiple rust diseases [13,15]. Among the ASR genes, *Lr9*, *Lr19*, *Lr24*, *Lr28*, *Lr29*, *Lr38*, *Lr47*, *Lr51*, and *Lr53* remain effective in many regions, whereas *Lr2c*, *Lr3*, *Lr16*, *Lr17*, *LrB*, *Lr3bg*, *Lr14b*, *Lr23*, and *Lr39* have largely lost effectiveness against most current races [6,10]. Due to its durability and broad-spectrum characteristics, APR has become a key focus in modern resistance breeding. However, its breeding application has been constrained by the complex genetic basis and minor individual effects of its component genes. Recent advances in genetic research and molecular marker technologies have enabled the mapping of over 200 QTLs for leaf rust resistance across diverse wheat populations, offering valuable resources for pyramiding breeding [16,17]. Widely grown resistant varieties such as Zhou 8425B and Pvon76 carry multiple APR genes, demonstrating the feasibility and value of this strategy in breeding programs [18].

With leaf rust posing an escalating threat under climate change, and given the concomitant challenges of resistance breakdown and linkage drag in existing genes, the ongoing discovery and deployment of novel resistance sources have become a pressing global imperative [19]. Therefore, identifying and cloning new genes, elucidating their genetic mechanisms, and developing efficient molecular tools for marker-assisted selection (MAS) represent critical tasks in current leaf rust resistance breeding [20]. In this study, we used a population of 253 recombinant inbred lines (RILs) derived from a cross between Liang66-S (moderate to high susceptibility to leaf rust) and Hengmai28 (high leaf rust resistance). The objectives were to: (1) identify novel leaf rust resistance loci and tightly linked SNP markers through genetic analysis; (2) develop user-friendly Kompetitive Allele Specific PCR (KASP) markers to facilitate efficient MAS for wheat leaf rust resistance.

## 2. Results

### 2.1. Phenotypic Analysis

**Figure 1:**
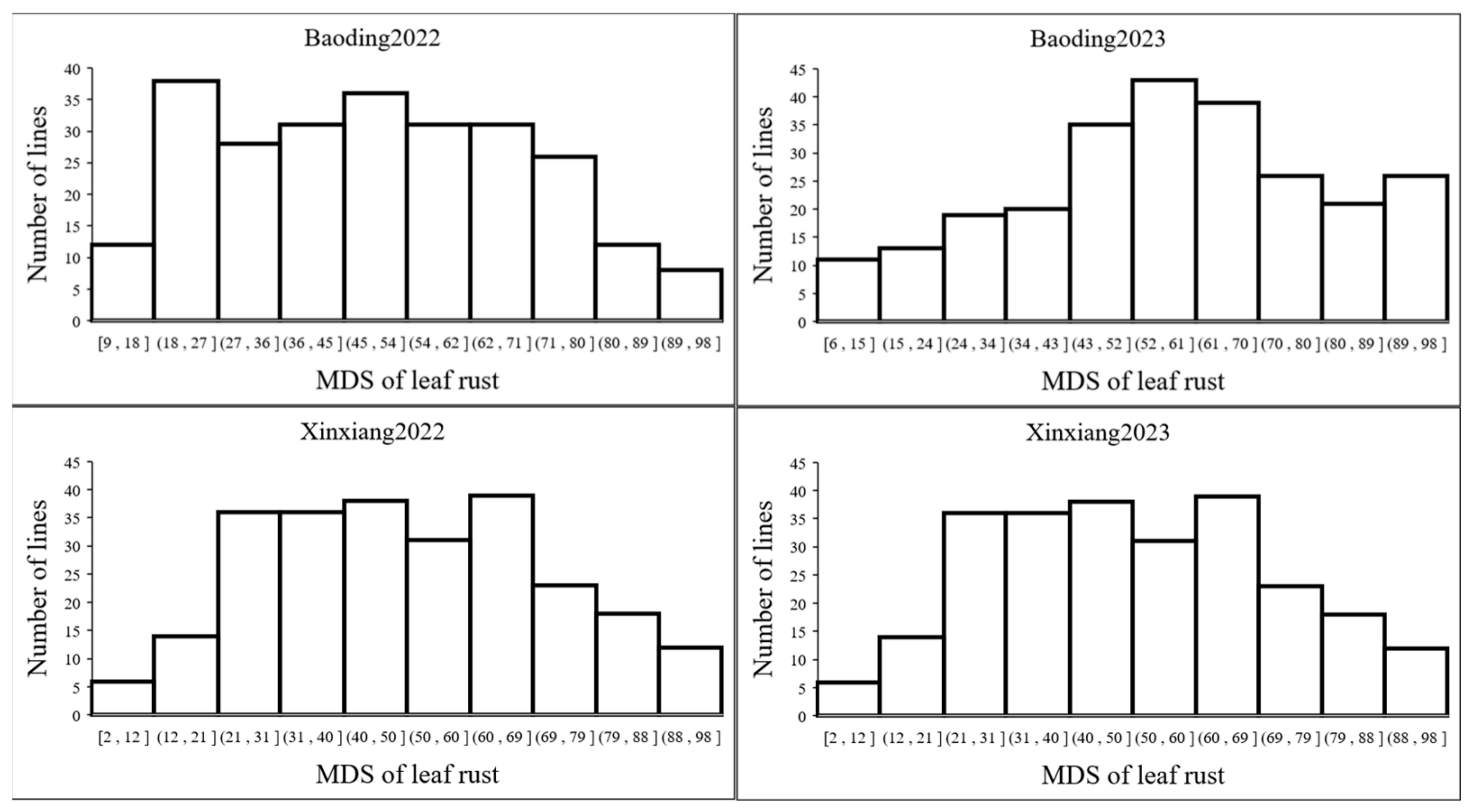
The distribution of MDS for leaf rust in the Liang66-S/Hengmai28 RIL population.

A RIL population consisting of 253 lines derived from the Liang66-S/Hengmai28 cross was evaluated for maximum disease severity (MDS) across four environments. Descriptive statistics revealed continuous variation with wide ranges (minimum 1.0–5.0, maximum 95.0–100.0) and mean values between 46.4 and 55.2, indicating moderate to high disease pressure (Table 1 and Figure 1). The standard deviations ranged from 20.6 to 22.4, and coefficients of variation (CV) were 40–46% across all environments, suggesting similar relative variability. The presence of transgressive segregation was evident as some RILs exhibited lower MDS than the resistant parent (Hengmai28) and others higher than the susceptible parent (Liang66-S). Frequency distributions were approximately normal, consistent with polygenic inheritance of adult-plant leaf rust resistance. Pairwise Pearson correlation coefficients among the four environments were moderately positive, ranging from 0.60 (Xinxiang2022 vs. Xinxiang2023) to 0.70 (Baoding2023 vs. Xinxiang2022 and Baoding2023 vs. Xinxiang2023) (Table A1). All correlations were highly significant (*p* < 0.05). These moderate correlation levels indicate that while the same genetic factors largely influenced disease severity across environments, genotype-by-environment (G × E) interactions were also present (Table A2).

Seedling inoculation results revealed that Liang66-S and Hengmai28 exhibited moderate to high susceptibility (infection type 3–4) to the leaf rust races THTT, PHTT, and THTS. Adult plant stage evaluations showed that Hengmai28 displayed high resistance (MDS 18–32% across all environments) to the leaf rust races, while Liang66-S exhibited moderate to high susceptibility (MDS = 42–58%). The analysis of variance showed that the genotype effect remained significant when tested against the genotype × environment interaction mean square (*p* = 0.001), while the genotype × environment interaction was highly significant (*p* < 0.001). Based on the estimated variance components (σ^2^_g_ = 21.39, σ^2^_ge_ = 241.37, σ^2^_e_ = 65.1), the broad-sense heritability (*H*_b_) on a line-mean basis was 0.25, indicating low-to-moderate genetic control. The genotype × environment interaction variance alone accounted for 73.6% of the total phenotypic variance (σ^2^_g_ + σ^2^_ge_ + σ^2^_e_), confirming that genotype performance was substantially environment-dependent.

### 2.2. QTL Mapping for Leaf Rust Resistance

**Figure 2:**
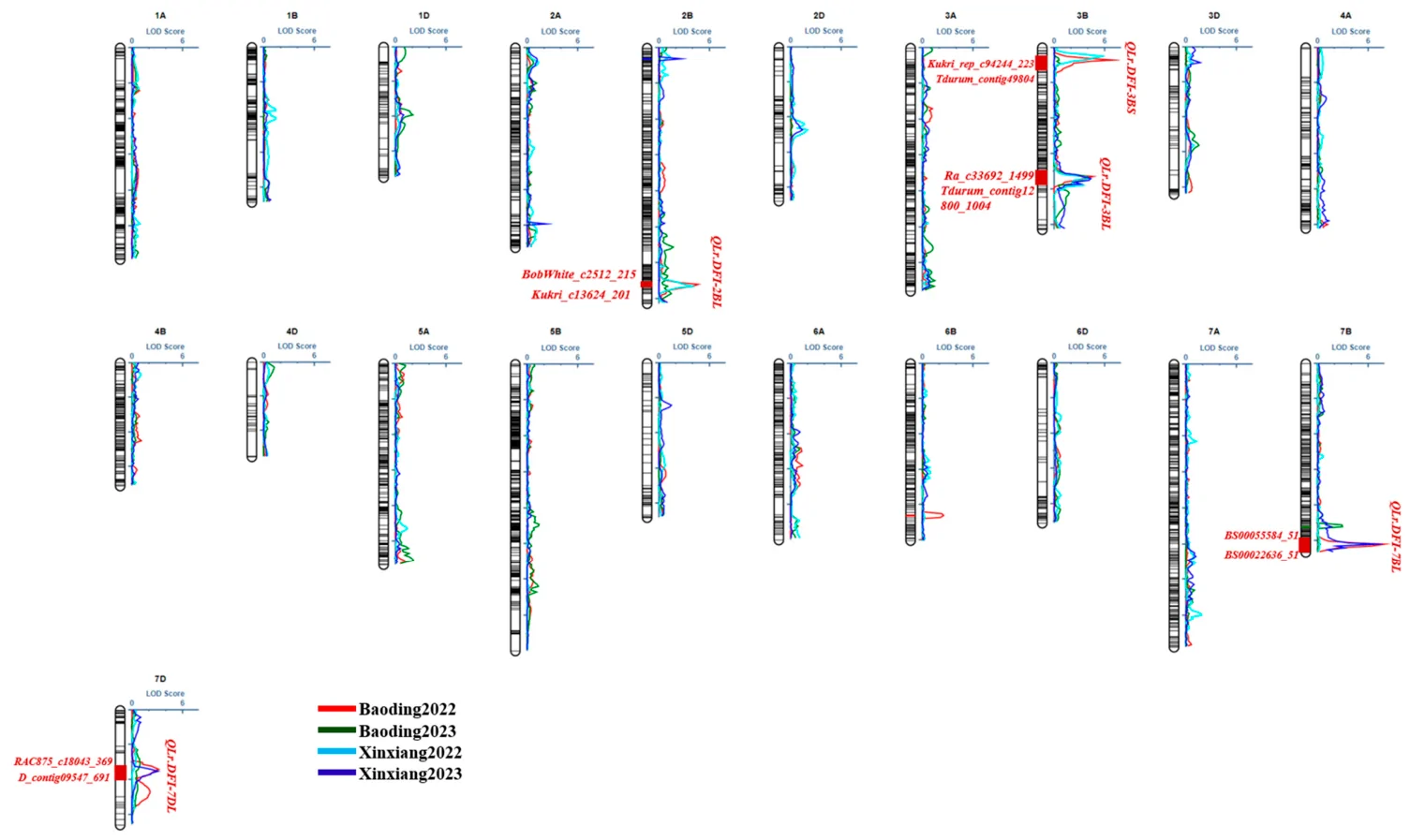
The QTL for leaf rust resistance identified in Liang66-S/Hengmai28 RIL population.

A total of five QTLs imparting APR to leaf rust were identified across four environments in the Liang66-S/Hengmai28 RIL population. These QTLs were distributed on chromosomes 2BL, 3BS, 3BL, 7BL, and 7DL (Table 2 and Figure 2). The phenotypic variation explained (R^2^) by each QTL ranged from 4.5% to 9.8%, and the LOD scores ranged from 2.9 to 7.9. On chromosome 2BL, a QTL designated *QLr.DFI-2BL* was detected in environments Baoding2022 and Xinxiang2022 with a 3.9-LOD of 4.6 and an R^2^ of 4.5–5.2% (additive effect: −3.8–−4.2). The resistance allele was contributed by Liang66-S. On chromosome 3B, two QTLs were identified. *QLr.DFI-3BS* was detected in Baoding2022 and Xinxiang2022 with an LOD of 5.9–7.0 and R^2^ of 7.7–8.5% (additive effect: 5.9–6.8), and mapping to chromosome 3BS with the resistance allele was contributed by Hengmai28. *QLr.DFI-3BL* was the most environmentally stable QTL in this study, being detected across all four environments (Baoding2022-Xinxiang2023). It exhibited an LOD of 3.8–4.5 and an R^2^ of 5.1–6.0% (additive effect: 5.0–5.8), with the resistance allele also derived from Hengmai28. On chromosome 7BL, *QLr.DFI-7BL* was the most robustly detected QTL in this study, with an LOD of 6.3–7.9 and an R^2^ of 8.4–9.8% (additive effect: 5.5–6.7). This QTL was identified in Baoding2022 and Xinxiang2023, with the resistance allele from Hengmai28. Finally, a minor *QTL QLr.DFI-7DL* was identified on chromosome 7D in Baoding2022 and Xinxiang2023, with an LOD of 2.9–3.2 and an R^2^ of 4.7–5.2% (additive effect: −3.8–−4.8). The resistance allele at this locus was contributed by Liang66-S.

### 2.3. Candidate Gene Analysis of Leaf Rust Resistance QTLs

**Figure 3:**
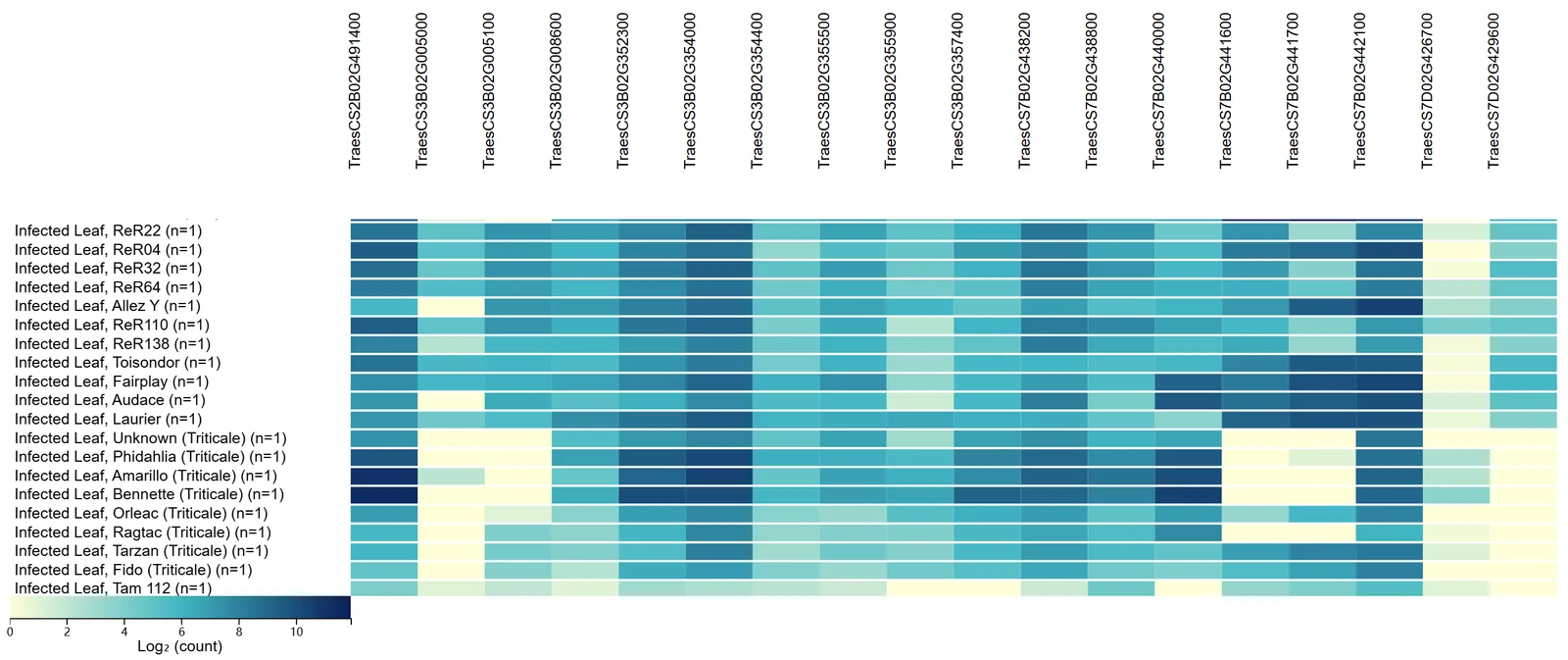
The expression pattern for the candidate genes for leaf rust resistance (https://www.wheat-expression.com/, accessed on 20 April 2026).

**Figure A1:**
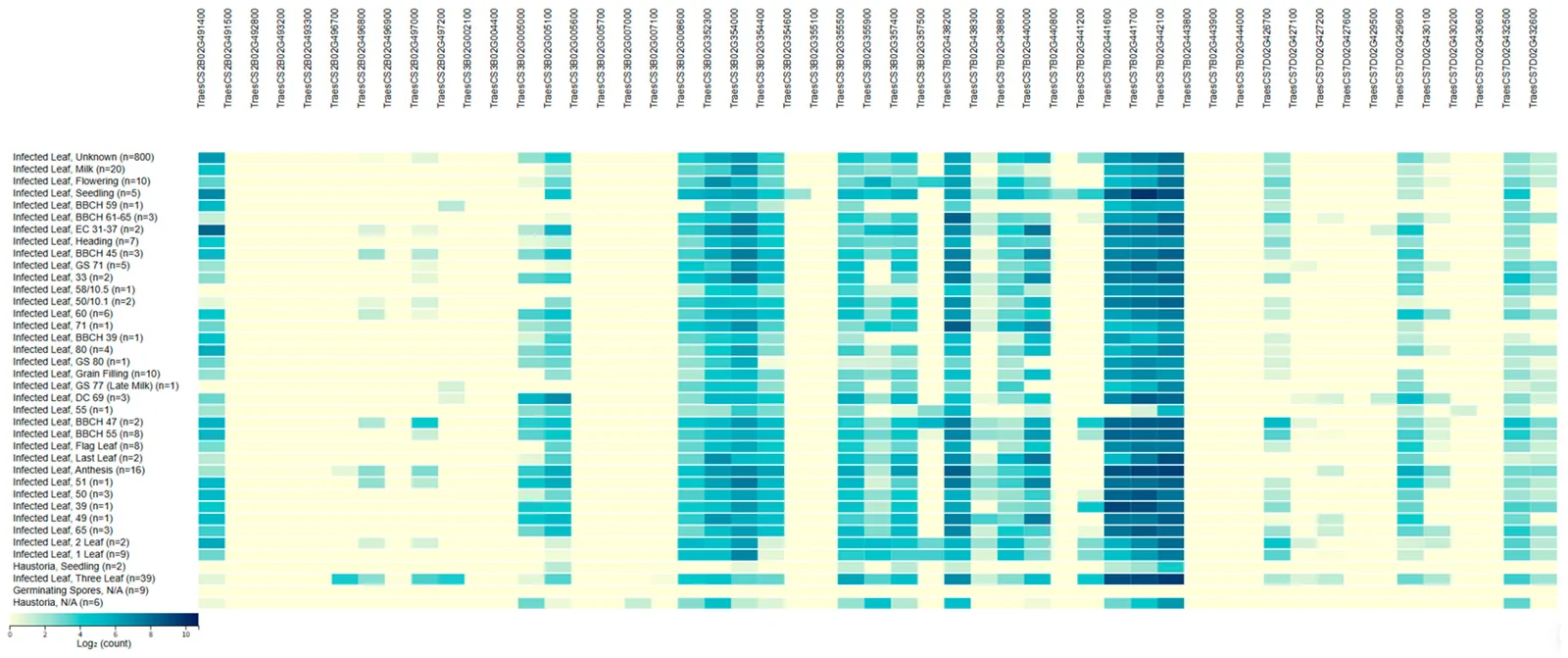
The expression pattern for the 51 candidate genes for leaf rust resistance located in the confidence interval (https://www.wheat-expression.com/, accessed on 11 April 2025).

To dissect the molecular basis of the five stable QTLs for wheat leaf rust resistance, we prioritized candidate genes involved in plant immunity based on four criteria: (i) high-confidence annotation from the IWGSC v1.0; (ii) functional annotation matching known immunity-related families (including NBS-LRR, RLK, CDPK, BTB/POZ, F-box, PPO, peroxidase, and transcription factors); (iii) leaf-preferential or stress-responsive expression patterns from the wheat-expression.com database; and (iv) physical positions within the QTL intervals delimited by flanking markers. Of the 51 genes initially identified (Table A3 and Figure A1), only 18 met the expression-based prioritization criteria (Table 3 and Figure 3).

For *QLr.DFI-2BL*, the physical interval contained several defense-related genes, including *TraesCS2B01G492800* (BTB/POZ-MATH), *TraesCS2B01G491500* (F-box), *TraesCS2B01G493200* and *TraesCS2B01G493300* (LRR-like protein), and multiple putative NBS-LRR disease resistance proteins (*TraesCS2B01G496700*, *TraesCS2B01G496800*, *TraesCS2B01G496900*, *TraesCS2B01G497000*, *TraesCS2B01G497200*). The potential BTB/POZ proteins have been implicated in immune signaling regulation in plants [21]. For *QLr.DFI-3BS*, the interval harbored *TraesCS3B01G002100* and *TraesCS3B01G004400* (F-box), *TraesCS3B01G005000* and *TraesCS3B01G005100* (RLKs), *TraesCS3B01G005600* (F-box), *TraesCS3B01G005700* (F-box), *TraesCS3B01G007000* and *TraesCS3B01G007100* (RLKs), and *TraesCS3B01G008600* (RLKs). For *QLr.DFI-3BL*, the QTL detected across all four environments contained *TraesCS3B01G352300* (serine/threonine-protein kinase), *TraesCS3B01G354000* (peroxidase), *TraesCS3B01G354400* (the putative NBS-LRR), *TraesCS3B01G354600* (CDPK), *TraesCS3B01G355100* (the putative BTB/POZ-MATH), *TraesCS3B01G355500* (F-box), *TraesCS3B01G355900* (PPO), *TraesCS3B01G357400* (ubiquitin-protein ligase), and *TraesCS3B01G357500* (ethylene-responsive factor-like transcription factor). The presence of a CDPK-like protein is noteworthy, as CDPKs are key Ca^2+^ decoders that activate downstream defense responses [22].

For *QLr.DFI-7BL*, which showed the strongest phenotypic effect, the interval included *TraesCS7B01G438200*, *TraesCS7B01G438300*, and *TraesCS7B01G438800* (ubiquitin system component Cue proteins), *TraesCS7B01G440000* (protein kinase), *TraesCS7B01G440800* (the putative TIR-NBS-LRR), *TraesCS7B01G441200* (RLKs), *TraesCS7B01G441600* (the putative NBS-LRR), *TraesCS7B01G441700* (the putative NBS-LRR), *TraesCS7B01G442100* (ubiquitin-protein ligase MARCH6), and *TraesCS7B01G443800*, *TraesCS7B01G443900*, *TraesCS7B01G444000* (MYB transcription factors). For *QLr.DFI-7DL*, the minor QTL contained *TraesCS7D01G426700* (ethylene-responsive transcription factor), *TraesCS7D01G427100*, *TraesCS7D01G427200*, *TraesCS7D01G427600*, *TraesCS7D01G430100*, *TraesCS7D01G430200*, *TraesCS7D01G432500*, *TraesCS7D01G432600* (F-box), *TraesCS7D01G429500* and *TraesCS7D01G429600* (the putative TIR-NBS-LRR), and *TraesCS7D01G430600* (the putative BTB/POZ). Collectively, these candidates span multiple layers of plant immunity, from pathogen recognition to downstream defense execution, suggesting that the resistance conferred by these QTLs is genetically complex.

To further prioritize the candidate genes within the five stable leaf rust resistance QTL, we examined their expression profiles using the public wheat expression database (wheat-expression.com) and retained those showing leaf-preferential or stress-responsive patterns. This filtering yielded 18 high-confidence genes distributed across all five QTL intervals. In *QLr.DFI-2BL*, *TraesCS2B01G491400* encoding PPO was notably expressed. The *QLr.DFI-3BS* interval contained three differentially expressed receptor-like kinases (*TraesCS3B01G005000*, *TraesCS3B01G005100*, *TraesCS3B01G008600*). The *QLr.DFI-3BL* QTL was consistently detected across all environments and harbored six expressed candidates: *TraesCS3B01G352300*, *TraesCS3B01G354000*, *TraesCS3B01G354400*, *TraesCS3B01G355500*, *TraesCS3B01G355900*, and *TraesCS3B01G357400*. For the major-effect QTL *QLr.DFI-7BL*, six highly expressed genes were identified: *TraesCS7B01G438200* and *TraesCS7B01G438800*, *TraesCS7B01G440000*, *TraesCS7B01G441600* and *TraesCS7B01G441700*, and *TraesCS7B01G442100*. In *QLr.DFI-7DL*, the expressed candidates included *TraesCS7D01G426700* and *TraesCS7D01G429600*.

### 2.4. Development and Validation of KASP Markers

**Figure 4:**
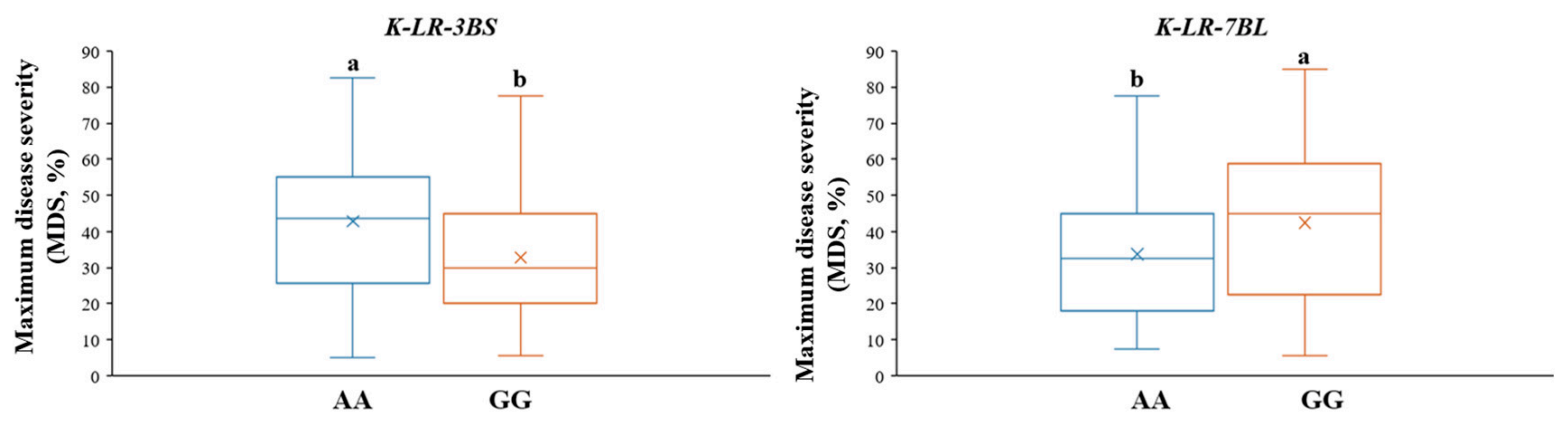
Validation of KASP markers *QLr.DFI-3BS* and *QLr.DFI-7BL* in a natural wheat population with 120 wheat accessions. Different letters indicate *p* < 0.05.

Five KASP markers were designed from the flanking sequences of the five stable QTLs (Table 4 and Table A4; Figure 4). These markers were genotyped in a natural validation panel comprising 120 diverse wheat varieties from the Huang-Huai region. The association between marker genotypes and leaf rust severity, measured by MDS, was evaluated across multiple environments. For *K-LR-3BS*, developed from *QLr.DFI-3BS*, varieties carrying the GG genotype (n = 59) exhibited significantly lower mean MDS of 32.8%, compared with 42.1% in those lines carrying the AA genotype (n = 47) (*p* < 0.05), confirming that the GG allele represents the resistant haplotype at this locus, consistent with the genetic effect observed in the mapping population (Table A5). Similarly, the marker *K-LR_7BL* developed from *QLr.DFI-7BL* on chromosome 7B revealed that varieties with the AA genotype (n = 52) displayed significantly reduced disease severity (33.7%) relative to those with the GG genotype (n = 45; 42.5%; *p* < 0.05), supporting the resistance-enhancing role of the AA allele, which was derived from the resistant parent Hengmai28. In addition, For *K-LR-3BS*, among the 120 accessions, 14 were heterozygous (AG), and 0 had missing data (failed calls); for *K-LR-7BL*, 16 were heterozygous (AG) and 7 had missing data. The call rates were 100% for *K-LR-3BS* and 94.2% for *K-LR-7BL*. For the remaining three markers: *K-LR_2BL* for *QLr.DFI-2BL* successfully genotyped the natural population but showed no significant association with MDS (*p* > 0.05); *K-LR_3BL* for *QLr.DFI-3BL* successfully genotyped the population but lacked significant association; and *K-LR_7DL* for *QLr.DFI-7DL* failed to produce reliable genotyping in the natural population. These results indicate that *K-LR-3BS* and *K-LR-7BL* are significantly associated with reduced leaf rust severity in the tested natural panel and represent promising markers for MAS. Given that only five targeted KASP markers were tested, no correction for multiple testing (e.g., Bonferroni or FDR) was applied. It should be noted that these associations were derived from a natural panel genotyped only for these five markers; therefore, population structure and relatedness could not be accounted for, and the results should be considered preliminary until confirmed with genome-wide marker data.

## 3. Discussion

Since the 1960s, CIMMYT has been engaged in breeding for APR and has successfully developed a set of multi-resistant varieties, such as Chapio, Cook, Pavon 76, Sonoita 81, and Tukuru, which have been widely adopted in developing countries [8]. In 2005, CIMMYT pyramided four to five adult plant resistance genes into single wheat varieties, generating APR materials with high levels of durable resistance. In recent years, developing APR varieties has become a major focus of international disease resistance breeding efforts [8]. Recently, rapid advances in genomics and molecular breeding technologies have provided powerful genetic tools for wheat breeders to dissect the genetic architecture of rust resistance [23]. QTL mapping, meta-analysis, and transcriptome profiling are now integrated to identify stable, environmentally robust loci [24]. In this context, mining resistance alleles from elite germplasm, developing functional markers, and applying marker-assisted or genomic selection to engineer novel germplasm are critical for advancing wheat breeding for enhanced leaf rust resistance.

In this study, although the phenotypic correlation coefficients among environments were moderately to highly positive (*r* = 0.60–0.70), the *H*_b_ was only 0.25, which is considered low to moderate. ANOVA revealed that the genotype main effect was significant (F = 1.33, *p* = 0.001) when tested against the G×E interaction, but this significance was substantially lower than when tested against the residual error, highlighting that much of the apparent genotypic differentiation was confounded by genotype-by-environment interactions. The G × E interaction itself was highly significant (F = 12.12, *p* < 0.001), indicating that genotype performance varied considerably across environments. Consistently, the estimated variance components showed that the G × E interaction (σ^2^_ge_ = 241.37) accounted for 73.6% of the total phenotypic variance, while genotypic variance (σ^2^_g_ = 21.39) contributed only about 6.5%. These results suggest that although genuine genetic differentiation exists among the RILs, the resistance in this population is strongly influenced by environmental fluctuations and G × E interactions. It should be noted that our experimental design (ANOVA) (Icimapping V4.1) does not separate additive from non-additive genetic effects; therefore, the reported genotypic variance should be interpreted as total heritable variation. In this study, we identified five APR QTLs for leaf rust resistance using an RIL population derived from Liang66-S × Hengmai28, which were evaluated across four environments. Among them, *QLr.DFI-3BS* and *QLr.DFI-7BL* consistently reduced disease seve rity in the RILs and were validated in an independent natural population using newly developed KASP markers *K-LR-3BS* and *K-LR_7BL*. These two QTLs, together with the three additional loci, provide valuable genetic resources for understanding the molecular basis of durable resistance.

### 3.1. Comparison with Previously Reported QTLs

*QLr.DFI-2BL* is located in a genomic region frequently associated with APR to leaf rust. Several independent studies have confirmed the presence of major QTLs for leaf rust APR on 2BL (Table A6). Zhang et al. [25] mapped a major APR QTL, *QLr.cau-2BL*, on 2BL between *IWB3854* (531.14 Mb) and *IWB21922* (616.48 Mb), and developed a diagnostic SSR marker (*Ta2BL_ssr7*) for MAS breeding. Buerstmayr et al. [26] also detected *QLr.ifa-2BL* (around 690.0 Mb) conferring both leaf rust and stripe rust resistance in the Austrian winter wheat cultivar Capo. Thus, *QLr.DFI-2BL* is a genuine and validated APR QTL, likely representing the same or a closely linked locus to previously reported 2BL QTLs. *QLr.DFI-3BS* is among the most consistently reported genomic regions for adult plant leaf rust resistance. AC Taber, a hard red spring wheat cultivar with long-lasting leaf rust resistance, was found to carry a QTL (around 33.1 Mb) on chromosome 3BS that maps to the same region as the adult plant resistance gene *Lr74* (tightly linked with *cfb5006* and *IWA6651***,** around 10–35 Mb) [27]. Furthermore, co-localized QTLs (5.0–13.5 Mb) for leaf rust and stripe rust on 3BS were also identified [27], indicating either closely linked genes or pleiotropic gene action. *QLr.DFI-3BS* is therefore judged as a genuine, validated QTL. Notably, its explained PVE is consistent with the moderate-effect QTLs commonly reported for this chromosome arm. *QLr.DFI-3BL* demonstrated the greatest environmental stability among all identified QTLs, being detected across all four environments. This stability suggests that this locus contributes to constitutive, broad-spectrum resistance. A novel leaf rust resistance QTLs (around 760 Mb) on 3BL in a wheat doubled haploid population, with the 3BL QTL conferring seedling resistance and different from the *QLr.DFI-3BL* identified in this study [28].

*QLr.DFI-7BL* is the most robust QTL in this study. The 7BL chromosome is a well-characterized genomic region harboring multiple rust resistance genes. The known slow-rusting gene *Lr68* (*Xgwm146*, *csSNP856*, *Xbarc32*, around 640–705 Mb) resides on 7BL and has been widely deployed in wheat breeding programs worldwide [9]. *Lr68* were mapped on chromosome 7B and developed tightly linked markers for MAS breeding [29]. Additionally, *QLr.usw-7BL* (nearly with *QLr.csiro-7B* at 730.1 Mb) was detected on the 7BL in the durum wheat Arnacoris, accounting for up to 65.9% of the disease severity variance [30]. CI13227, a wheat accession with slow-rusting resistance, was also reported to carry a QTL on 7BL (around 640 Mb) explaining 6.8% to 28.5% of the phenotypic variance [31]. The 7BL QTL has also been shown to confer simultaneous resistance to both leaf rust and stripe rust [26]. Given its strong linkage to established resistance loci and high LOD value, *QLr.DFI-7BL* is considered a genuine, potentially major APR QTL. Finally, *QLr.DFI-7DL* resides in a genomic region known to carry important rust resistance genes. The long arm of chromosome 7D harbors the well-characterized leaf rust resistance gene *Lr19* (*XsdauK3734* and *XsdauK2839* around 650–712.0 Mb), which was originally transferred from *Thinopyrum ponticum* and has been widely used in wheat breeding [32]. Although *QLr.DFI-7DL* is detected as a minor QTL in this study, its chromosomal location on 7DL supports its authenticity. Further fine-mapping will be necessary to determine whether *QLr.DFI-7DL* corresponds to *Lr19* or represents a different resistance locus on 7DL (Table A3).

Among the identified QTLs, *QLr.DFI-2BL*, *QLr.DFI-3BS*, and *QLr.DFI-7BL* are considered previously reported and validated loci as they consistently map to genomic regions frequently associated with APR to leaf rust and have been documented in multiple independent studies. In contrast, *QLr.DFI-3BL* and *QLr.DFI-7DL* are regarded as new: *QLr.DFI-3BL* was reported as a novel QTL [28], while *QLr.DFI-7DL*, though residing on a chromosome arm known to carry *Lr19*, requires further fine-mapping to determine its relationship to known resistance genes and is detected as a minor QTL in this study, suggesting it may represent a distinct or uncharacterized locus. In summary, the most robust QTLs are *QLr.DFI-3BL* and *QLr.DFI-7BL*, which should be prioritized for further fine-mapping and potential MAS breeding.

### 3.2. Candidate Gene Functions and Resistance Mechanisms

In this study, we identified 18 high-confidence candidate genes within the five QTL intervals, encompassing classical resistance genes, signaling components, and metabolic enzymes involved in diverse immune pathways. Canonical NBS-LRR genes were found in *QLr.DFI-3BS*, *QLr.DFI-7BL*, and *QLr.DFI-2BL*, consistent with the fact that most major R genes encode putative NBS-LRR proteins [7]. Notably, *QLr.DFI-7BL* contains two adjacent NBS-LRR genes (*TraesCS7B01G441600* and *TraesCS7B01G441700*), reminiscent of paired NLRs recently shown to function cooperatively in wheat stripe rust resistance [33]. Beyond canonical R genes, we identified several regulatory and metabolic candidates. A CDPK gene (*TraesCS3B01G354600*) within *QLr.DFI-3BL* encodes a key Ca^2+^ decoder that activates downstream defense responses, and multiple wheat CDPKs are induced by leaf rust infection [22,24]. BTB/POZ-MATH domain proteins, involved in ubiquitin-proteasome regulation and R-protein stability [34], were detected in three independent QTLs (*QLr.DFI-2BL*, *-3BL*, and *-7DL*), while F-box proteins, also part of the ubiquitin ligase complex, appeared in multiple intervals (*QLr.DFI-2BL*, *-3BS*, *-3BL*, *-7DL*), further underscoring the importance of ubiquitination in tuning defense signaling [21,35]. Oxidative-burst enzymes—polyphenol oxidases (PPO), peroxidases, and germin-like proteins (GLP), were located in specific QTLs (*TraesCS2B01G491400* in *QLr.DFI-2BL*; *TraesCS3B01G354000* peroxidase and *TraesCS3B01G355900* PPO of *QLr.DFI-3BL*; *TraesCS7B01G442000* GLP in *QLr.DFI-7BL*), and their leaf-biased expression supports a role in reactive oxygen species generation and cell wall reinforcement against biotrophic fungi, including *Puccinia* species [36,37]. In addition, multiple LRR-RLKs (e.g., *TraesCS2B01G493200*, *-493300*, *TraesCS3B01G007100*) and ethylene-responsive factors (ERFs) suggest that both PAMP-triggered immunity (PTI) and hormone signaling are integrated into the resistance network defined by these QTLs [24]. Overall, the 18 prioritized candidates, which show significant expression in leaf tissues or under stress conditions, provide strong functional relevance to the leaf rust resistance conferred by these QTLs. The convergence of expression evidence with known immune functions justifies future functional validation through virus-induced gene silencing (VIGS) or genome editing, with particular emphasis on the paired NBS-LRR genes in *QLr.DFI-7BL* and the multi-component signaling network in *QLr.DFI-3BL*, to elucidate their precise roles and facilitate marker-assisted breeding for durable leaf rust resistance in wheat.

### 3.3. Limitations and Future Perspectives

Despite the identification of five QTLs and the validation of two KASP markers, several limitations of this study should be acknowledged, including the moderate population size and marker density that resulted in large confidence intervals, the lack of functional characterization for candidate genes, and the limited environmental and population breadth for marker validation. Critically, because the natural diversity panel was genotyped exclusively for the five target KASP markers derived from previous QTL mapping, we were unable to correct for population stratification or familial relatedness, which may confound the observed marker–trait associations. Therefore, while *K-LR-3BS* and *K-LR-7BL* showed statistically significant associations, they should be regarded as preliminarily validated rather than fully diagnostic. The stability of these associations should be further tested in broader germplasm sets using genome-wide markers with proper correction for population structure. To address these genetic and mechanistic gaps, high-resolution fine mapping of *QLr.DFI-3BS* and *QLr.DFI-7BL* using large segregating populations is required to narrow candidate intervals to a few hundred kilobases, followed by positional cloning coupled with transcriptomic profiling and gene silencing/overexpression in wheat to identify and validate causal genes. The recently available wheat pan-genome resources and multi-omics approaches will greatly accelerate the cloning of adult plant resistance genes [23,38,39,40]. Ultimately, integrating validated diagnostic markers for these QTLs into genomic selection models, combined with marker-assisted pyramiding of other cloned APR genes (e.g., *Lr34*, *Lr46*, *Lr68*, and *Lr74*), will enable the development of wheat cultivars with durable, broad-spectrum leaf rust resistance in the Huang-Huai region and beyond.

### 3.4. Perspectives for Adult-Plant Leaf Rust Resistance Breeding

The two validated KASP markers provide immediate, cost-effective tools for integrating leaf rust resistance into winter wheat varieties adapted to the Huang-Huai region. Their direct application in MAS enables breeders to efficiently select progenies carrying favorable alleles without time-consuming field phenotyping. Critically, the resistance alleles at *QLr.DFI-3BS* and *QLr.DFI-7BL* are derived from the resistant parents used in our RIL population, which can serve as valuable donor germplasm for crossing programs. By crossing these donors with elite but susceptible cultivars, followed by marker-assisted backcrossing, breeders can rapidly transfer the QTLs into adapted backgrounds while retaining agronomic performance. For durable resistance, the validated markers should be multiplexed with other known resistance genes, particularly the widely deployed adult-plant genes *Lr34* and *Lr46*, as additive or synergistic effects have been reported [8,12]. Moreover, the integration of *K-LR-3BS* and *K-LR_7BL* into a low-density, breeder-friendly genotyping panel would allow routine screening of early-generation populations, enabling the selection of resistant individuals before field evaluation and significantly reducing breeding cycle time. Beyond immediate MAS applications, the two QTL regions offer promising targets for positional cloning and candidate gene validation; the validated markers can serve as anchor points for fine-mapping and for developing more tightly linked markers to further enhance selection precision. Collectively, our findings not only enrich the genetic resources for leaf rust resistance but also provide actionable tools to accelerate the development of resistant cultivars, thereby contributing to sustainable wheat production in the face of increasing disease pressure.

## 4. Materials and Methods

### 4.1. Plant Materials and Phenotypic Identification

The parental line Liang66-S exhibited adult-plant leaf rust responses varying between moderate susceptibility and moderate resistance, whereas Hengmai28 displayed levels ranging from moderate to high resistance. A recombinant inbred line (RIL) population consisting of 253 F_2:8_ lines, derived from a cross between Liang66-S and Hengmai28, was used for field experiments. This population was grown during the 2021–2022 and 2022–2023 growing seasons at two locations: Xinxiang (35°18′ N, 113°52′ E, Henan Province) and Baoding (38°52′ N, 115°28′ E, Hebei Province). The field trial employed a randomized complete block design with three replications, and each plot row was 3 m long. The susceptible cultivar Zhengzhou 5389 served as both the disease control and the inoculum spreader; one spreader row was planted after every ten rows of test material to guarantee uniform inoculation. To validate the genetic effects of the leaf rust resistance quantitative trait loci (QTLs) identified, a natural panel comprising 120 wheat accessions originating from the Huang-Huai wheat region was employed. This validation panel was planted during the 2020–2021 and 2021–2022 growing seasons at Baoding (38°52′ N, 115°28′ E, Hebei Province) and Zhengzhou (35°44′ N, 113°42′ E, Henan Province), also following a randomized complete block design with three replicates, a row length of 2 m, and a row spacing of 20 cm.

Xinxiang and Zhengzhou in Henan, together with Baoding in Hebei, are endemic regions for wheat leaf rust, and the predominant, prevailing physiological races in these areas are THTT, PHTT, and THTS [41]. At the wheat jointing stage, urediniospores of the three races were mixed in equal proportions and suspended in 0.03% Tween-20 solution for spray inoculation. Approximately four weeks after inoculation, when the leaves of the susceptible check Zhengzhou 5389 were nearly fully covered with uredinia, disease severity assessments were conducted on the mapping population. Field evaluations were performed weekly, recording the leaf rust severity of both parents and all progeny lines, using the percentage of leaf area covered by uredinia as the scoring criterion. In each environment, the disease assessment was repeated two to three times, and the MDS was retained as the phenotypic data for subsequent linkage analysis [42].

### 4.2. Statistical Analysis and Linkage Mapping

Analysis of variance (ANOVA) was performed using IciMapping v4.2 (http://www.isbreeding.net/, accessed on 25 December 2025) [43], and the Pearson correlation coefficient was used to evaluate the correlation of maximum disease severity across different environments. *H_b_*^2^ was estimated as *H_b_*^2^ = σ^2^g/σ^2^p, where σ^2^g is the genotypic variance and σ^2^p is the phenotypic variance (σ^2^p = σ^2^g + σ^2^ge + σ^2^e), with σ^2^ge and σ^2^e representing genotype-by-environment interaction variance and residual (environmental) variance, respectively. Variance components were estimated from the ANOVA mean squares using the expected mean square method. For the RIL population evaluated across four environments (e = 4) with three replicates per environment (r = 3), the components were derived as follows: σ^2^_g_ = (MS_g_ − MS_ge_)/(e × r) = (1045.9 − 789.2)/12 = 21.39, σ^2^_ge_ = (MS_ge_ − MS_e_)/r = (789.2 − 65.1)/3 = 241.37, σ^2^e = MS_e_ = 65.1. *H*_b_ on a line-mean basis was calculated as: *H*_b_ = σ^2^_g_/(σ^2^_g_ + σ^2^g_e_/e + σ^2^_e_/(e × r)) = 21.39/(21.39 + 60.34 + 5.43) ≈ 0.25. All calculations are based on the variance components derived from the corrected ANOVA (Table A2).

Genotyping of Liang66-S, Hengmai28, and all RIL lines was performed using the wheat 90K SNP array [44]. The genetic map was constructed [45], with 6526 high-quality polymorphic markers. The inclusive composite interval mapping (ICIM) method implemented in IciMapping v4.2 was used to perform QTL mapping for maximum disease severity of the Liang66-S/Hengmai28 RIL population across four environments. This method combines the advantages of interval mapping and multiple regression analysis. QTLs were mapped separately for each environment using the ICIM method, and the LOD and R^2^ values are presented in Table 2. A 1000-permutation test was performed to determine the significance threshold (*p* = 0.05), with an LOD threshold of 2.6 and a step size of 1 cM, to generate LOD curves and identify QTL positions. In this study, a QTL detected in two or more environments was defined as a stable QTL. In this study, “DFI” denotes the Dryland Farming Institute, Hebei Academy of Agriculture and Forestry Sciences, and our QTL naming follows the standard wheat convention (QTL + trait abbreviation + “DFI” + chromosome).

### 4.3. Development of KASP Markers

KASP primers were designed using the flanking and internal markers of the identified QTLs for leaf rust resistance. Primer design was carried out via the PolyMarker web interface (http://www.polymarker.info/, accessed on 15 April 2026) [46], and all primer sequences are provided in Table A4. Each KASP primer set comprises two allele-specific competing primers and a single common primer, with only one nucleotide difference at the 3′ end of the two competing primers. After selecting locus-specific or semi-specific primers, the 5′ ends of the two competing primers were separately tagged with FAM and HEX sequences. All primers were synthesized by Sangon Biotech Co., Ltd. (Shanghai, China). The KASP PCR reaction mixture contained: 2.0 µL of 2 × KASP Master Mix, 0.0336 µL of mixed KASP primers, and 2 µL of template DNA (50 ng/µL). Amplification was conducted using a water-bath PCR system from LGC Limited (UK). The thermal cycling conditions were: one cycle of pre-denaturation at 94 °C for 15 min; followed by 10 touchdown cycles consisting of denaturation at 94 °C for 20 s and annealing for 1 min per cycle, with the annealing temperature decreasing from 65 °C to 57 °C; and finally 35 cycles of denaturation at 94 °C for 20 s and annealing at 57 °C for 1 min. Following amplification, fluorescence intensity was measured using a PHEstar plate reader (BMG Labtech GmbH, Germany) with the 384-well KASP scanning program. Genotyping data were analyzed using KLUSTER v1.0 (LGC, London, UK). A two-tailed Welch’s *t*-test to compare the mean MDS between the two homozygous genotype classes (e.g., AA vs. GG) for each marker. For the natural panel, the MDS used for association is the average MDS across the two evaluation years (2020–2021 and 2021–2022) at both Baoding and Zhengzhou. Heterozygous calls (e.g., AG) were not included in the *t*-test comparisons between the two homozygous classes because their phenotypic expectation is ambiguous. The number of heterozygous accessions is reported alongside the call rate for complete transparency. Because only the five KASP markers were genotyped in this natural panel and genome-wide SNP profiles were not obtained, formal correction for population structure and relatedness could not be performed. For the five KASP markers tested, no adjustment for multiple comparisons was performed as these markers were chosen as targeted candidates from prior QTL mapping rather than as a genome-wide screen.

### 4.4. Candidate Gene Prediction

Candidate genes were identified based on IWGSC v1.0 [47]. Candidate genes were selected based on the physical interval defined by the flanking markers of each QTL, using the Chinese Spring reference genome (IWGSC v1.0). The criteria for candidate gene selection followed: (i) high-confidence genes located within the mapped QTL interval based on IWGSC v1.0 annotation; (ii) genes with protein domains/functional annotations consistent with known disease resistance genes (e.g., NBS-LRR, RLK, CDPK, BTB/POZ); (iii) genes showing leaf-preferred expression and/or stress-responsive expression patterns based on public database (wheat-expression.com) [48,49], focusing on expression patterns across different developmental stages and under stress conditions relevant to disease response.

## 5. Conclusions

Wheat leaf rust seriously threatens wheat production and causes significant losses to both yield and quality. Breeding resistant cultivars is currently the most economically and environmentally sustainable strategy for leaf rust control. In this study, five APR QTLs for leaf rust were identified in the Liang66-S/Hengmai28 RIL population, and they explained 4.5–9.8% of the PVE. Two developed KASP markers (*K-LR-3BS* and *K-LR_7BL*) were validated in a panel of 120 Chinese wheat cultivars and confirmed to be significantly associated with leaf rust resistance, whereas the remaining three markers, *K-LR_2BL* for *QLr.DFI-2BL*, *K-LR_3BL* for *QLr.DFI-3BL*, and *K-LR_7DL* for *QLr.DFI-7DL*, either showed no significant association with MDS (*p* > 0.05) or failed to produce reliable genotyping in the natural population. Both *K-LR-3BS* and *K-LR-7BL* are promising candidates for leaf rust resistance, but their routine application in MAS awaits independent validation in diverse genetic backgrounds and with proper control for population structure. Furthermore, multiple candidate genes involved in various immune pathways were identified based on public gene expression data, including NBS-LRR, CDPK, BTB/POZ, F-box, PPO, peroxidase, and ERF. The functional QTLs, breeding-applicable KASP markers, and candidate gene resources obtained in this study provide important support for molecular marker-assisted selection for wheat leaf rust resistance breeding.

## Figures and Tables

**Table 1:** Descriptive statistics for leaf rust maximum disease severity (MDS) in the Liang66-S/Hengmai28 RIL population across four environments.

MDS	E1	E2	E3	E4
Min	1.0	2.0	3.0	5.0
Max	99.0	100.0	95.0	98.0
Mean	48.4	55.1	46.4	50.5
Std	20.6	22.1	21.4	22.4
CV	0.43	0.40	0.46	0.44

E1: Baoding2022; E2: Baoding2023; E3: Xinxiang2022; E4: Xinxiang2023.

**Table 2:** Quantitative trait loci for leaf rust resistance identified in the Liang66-S/Hengmai28 RIL population.

QTL	Chr.	Environment	Left Marker	Start (Mb)	Right Marker	End (Mb)	Add	LOD	R^2^
*QLr.DFI-2BL*	2B	E1h/E3danlan	*BobWhite_c2512_215*	689.9	*Kukri_c13624_201*	692.5	−3.8–−4.2	3.9–4.6	4.5–5.2
*QLr.DFI-3BS*	3B	E1/E3	*Kukri_rep_c94244_223*	2.3	*Tdurum_contig49804_392*	4.1	5.9–6.8	5.9–7.0	7.7–8.5
*QLr.DFI-3BL*	3B	E1/E2lvse/E3/E4shenzhen	*Ra_c33692_1499*	562.4	*Tdurum_contig12800_1004*	567.2	5.0–5.8	3.8–4.5	5.1–6.0
*QLr.DFI-7BL*	7B	E1/E4	*BS00055584_51*	704.6	*BS00022636_51*	708.1	5.5–6.7	6.3–7.9	8.4–9.8
*QLr.DFI-7DL*	7D	E1/E4	*D_contig09547_691*	547.3	*RAC875_c18043_369*	550.1	−3.8–−4.8	2.9–3.2	4.7–5.2

E1: Baoding2022; E2: Baoding2023; E3: Xinxiang2022; E4: Xinxiang2023.

**Table 3:** The 18 prioritized candidate genes within the leaf rust resistance QTL intervals in the Liang66-S/Hengmai28 RIL population.

QTL	Candidate Gene v1.0	Candidate Gene v1.1	Annotation
*QLr.DFI-2BL*	*TraesCS2B01G491400*	*TraesCS2B02G491400*	Polyphenol oxidase
*QLr.DFI-3BS*	*TraesCS3B01G005000*	*TraesCS3B02G005000*	receptor kinase 1
*QLr.DFI-3BS*	*TraesCS3B01G005100*	*TraesCS3B02G005100*	receptor kinase 1
*QLr.DFI-3BS*	*TraesCS3B01G008600*	*TraesCS3B02G008600*	Receptor-like kinase
*QLr.DFI-3BL*	*TraesCS3B01G352300*	*TraesCS3B02G352300*	Serine/threonine-protein kinase
*QLr.DFI-3BL*	*TraesCS3B01G354000*	*TraesCS3B02G354000*	Peroxidase
*QLr.DFI-3BL*	*TraesCS3B01G354400*	*TraesCS3B02G354400*	Disease resistance protein (NBS-LRR class)
*QLr.DFI-3BL*	*TraesCS3B01G355500*	*TraesCS3B02G355500*	F-box family protein
*QLr.DFI-3BL*	*TraesCS3B01G355900*	*TraesCS3B02G355900*	Polyphenol oxidase
*QLr.DFI-3BL*	*TraesCS3B01G357400*	*TraesCS3B02G357400*	E3 ubiquitin-protein ligase
*QLr.DFI-7BL*	*TraesCS7B01G438200*	*TraesCS7B02G438200*	Ubiquitin system component Cue protein
*QLr.DFI-7BL*	*TraesCS7B01G438800*	*TraesCS7B02G438800*	Ubiquitin system component Cue protein
*QLr.DFI-7BL*	*TraesCS7B01G440000*	*TraesCS7B02G440000*	Protein kinase, putative
*QLr.DFI-7BL*	*TraesCS7B01G441600*	*TraesCS7B02G441600*	The putative NBS-LRR resistance-like protein
*QLr.DFI-7BL*	*TraesCS7B01G441700*	*TraesCS7B02G441700*	The putative Disease resistance protein (NBS-LRR class)
*QLr.DFI-7BL*	*TraesCS7B01G442100*	*TraesCS7B02G442100*	E3 ubiquitin-protein ligase MARCH6
*QLr.DFI-7DL*	*TraesCS7D01G426700*	*TraesCS7D02G426700*	Ethylene-responsive transcription factor
*QLr.DFI-7DL*	*TraesCS7D01G429600*	*TraesCS7D02G429600*	Putative disease resistance protein (TIR-NBS-LRR class)

**Table 4:** Validation of KASP markers in a natural wheat population with 120 accessions.

KASP	QTL	Genotype	Number of Varieties	MDS (%)	*p* Value	Call Rate (%)
*K-LR-3BS*	*QLr.DFI-3BS*	AA	47	42.1	0.0092	100
		GG	59	32.8		
		AG	14	38.9		
		Missing/Failed	0			
*K-LR-7BL*	*QLr.DFI-7BL*	AA	52	33.7	0.021	94.2
		GG	45	42.5		
		AG	16	31.4		
		Missing/Failed	7	42.1		

## Data Availability

All of the data generated or analyzed during this study are included in this published article.

## Abbreviations

APR	Adult-plant resistance
ASR	All-stage resistance
ICIM	Inclusive composite interval mapping
KASP	Kompetitive allele-specific PCR
LOD	Logarithm of odds
MDS	Maximum disease severity
PVE	Phenotypic variation explained
QTL	Quantitative trait loci
RIL	Recombinant inbred line
SNP	Single nucleotide polymorphism

## Appendix A

**Table A1:** Pearson correlation coefficients of leaf rust MDS among four environments in Liang66-S/Hengmai28 RIL population.

Correlation Coefficients	E1	E2	E3	E4
E1	1.0			
E2	0.68	1.0		
E3	0.70	0.70	1.0	
E4	0.66	0.70	0.66	1.0

E1: Baoding2022; E2: Baoding2023; E3: Xinxiang2022; E4: Xinxiang2023.

**Table A2:** ANOVA for the MDS of leaf rust in the Liang66-S/Hengmai28 RIL population across four environments.

Source of Variation	DF	MS	F Value	*p*-Value
Environment	3	3676.3	56.5 ***	*p* < 0.001 ***
Genotype	252	1045.9	1.33 ***	*p* = 0.001 **
Genotype ×Environment	756	789.2	12.12 ***	*p* < 0.001 ***
Replicate (Environment)	8	37.9	0.58	n.s.
Error	2016	65.1		

F-values for Genotype and Genotype × Environment were tested against the Genotype × Environment mean square and the Error mean square, respectively. Exact *p*-values: Genotype, *p* = 0.001; Genotype × Environment, *p* < 0.001. ** means significant at *p* < 0.01; *** means significant at *p* < 0.001.

**Table A3:** A total of 51 candidate genes within the leaf rust resistance QTL intervals in Liang66-S/Hengmai28 RIL population.

QTL	Candidate Gene v1.0	Candidate Gene v1.1	Annotation
*QLr.DFI-2BL*	*TraesCS2B01G491400*	*TraesCS2B02G491400*	Polyphenol oxidase
*QLr.DFI-2BL*	*TraesCS2B01G491500*	*TraesCS2B02G491500*	F-box family protein
*QLr.DFI-2BL*	*TraesCS2B01G492800*	*TraesCS2B02G492800*	Putative BTB/POZ and MATH domain-containing protein
*QLr.DFI-2BL*	*TraesCS2B01G493200*	*TraesCS2B02G493200*	Leucine-rich repeat receptor-like protein
*QLr.DFI-2BL*	*TraesCS2B01G493300*	*TraesCS2B02G493300*	Leucine-rich repeat receptor-like protein
*QLr.DFI-2BL*	*TraesCS2B01G496700*	*TraesCS2B02G496700*	The putative NBS-LRR disease resistance protein-like protein
*QLr.DFI-2BL*	*TraesCS2B01G496800*	*TraesCS2B02G496800*	disease resistance protein (TIR-NBS-LRR class
*QLr.DFI-2BL*	*TraesCS2B01G496900*	*TraesCS2B02G496900*	Disease resistance protein (TIR-NBS-LRR class)
*QLr.DFI-2BL*	*TraesCS2B01G497000*	*TraesCS2B02G497000*	The putative NBS-LRR disease resistance protein-like protein
*QLr.DFI-2BL*	*TraesCS2B01G497200*	*TraesCS2B02G497200*	Putative NBS-LRR disease resistance protein-like protein
*QLr.DFI-3BS*	*TraesCS3B01G002100*	*TraesCS3B02G002100*	F-box like protein
*QLr.DFI-3BS*	*TraesCS3B01G004400*	*TraesCS3B02G004400*	F-box family protein
*QLr.DFI-3BS*	*TraesCS3B01G005000*	*TraesCS3B02G005000*	receptor kinase 1
*QLr.DFI-3BS*	*TraesCS3B01G005100*	*TraesCS3B02G005100*	receptor kinase 1
*QLr.DFI-3BS*	*TraesCS3B01G005600*	*TraesCS3B02G005600*	Receptor-like kinase
*QLr.DFI-3BS*	*TraesCS3B01G005700*	*TraesCS3B02G005700*	F-box family protein
*QLr.DFI-3BS*	*TraesCS3B01G007000*	*TraesCS3B02G007000*	Receptor-like kinase
*QLr.DFI-3BS*	*TraesCS3B01G007100*	*TraesCS3B02G007100*	Leucine-rich repeat receptor-like protein
*QLr.DFI-3BS*	*TraesCS3B01G008600*	*TraesCS3B02G008600*	Receptor-like kinase
*QLr.DFI-3BL*	*TraesCS3B01G352300*	*TraesCS3B02G352300*	Serine/threonine-protein kinase
*QLr.DFI-3BL*	*TraesCS3B01G354000*	*TraesCS3B02G354000*	Peroxidase
*QLr.DFI-3BL*	*TraesCS3B01G354400*	*TraesCS3B02G354400*	Disease resistance protein (NBS-LRR class)
*QLr.DFI-3BL*	*TraesCS3B01G354600*	*TraesCS3B02G354600*	calcium-dependent kinase-like protein
*QLr.DFI-3BL*	*TraesCS3B01G355100*	*TraesCS3B02G355100*	Putative BTB/POZ and MATH domain-containing protein
*QLr.DFI-3BL*	*TraesCS3B01G355500*	*TraesCS3B02G355500*	F-box family protein
*QLr.DFI-3BL*	*TraesCS3B01G355900*	*TraesCS3B02G355900*	Polyphenol oxidase
*QLr.DFI-3BL*	*TraesCS3B01G357400*	*TraesCS3B02G357400*	E3 ubiquitin-protein ligase
*QLr.DFI-3BL*	*TraesCS3B01G357500*	*TraesCS3B02G357500*	Ethylene-responsive factor-like transcription factor
*QLr.DFI-7BL*	*TraesCS7B01G438200*	*TraesCS7B02G438200*	Ubiquitin system component Cue protein
*QLr.DFI-7BL*	*TraesCS7B01G438300*	*TraesCS7B02G438300*	Ubiquitin system component Cue protein
*QLr.DFI-7BL*	*TraesCS7B01G438800*	*TraesCS7B02G438800*	Ubiquitin system component Cue protein
*QLr.DFI-7BL*	*TraesCS7B01G440000*	*TraesCS7B02G440000*	Protein kinase, putative
*QLr.DFI-7BL*	*TraesCS7B01G440800*	*TraesCS7B02G440800*	Putative Disease resistance protein (TIR-NBS-LRR class)
*QLr.DFI-7BL*	*TraesCS7B01G441200*	*TraesCS7B02G441200*	Receptor protein kinase-like protein
*QLr.DFI-7BL*	*TraesCS7B01G441600*	*TraesCS7B02G441600*	The putative NBS-LRR resistance-like protein
*QLr.DFI-7BL*	*TraesCS7B01G441700*	*TraesCS7B02G441700*	The putative Disease resistance protein (NBS-LRR class)
*QLr.DFI-7BL*	*TraesCS7B01G442100*	*TraesCS7B02G442100*	E3 ubiquitin-protein ligase MARCH6
*QLr.DFI-7BL*	*TraesCS7B01G443800*	*TraesCS7B02G443800*	MYB transcription factor
*QLr.DFI-7BL*	*TraesCS7B01G443900*	*TraesCS7B02G443900*	MYB transcription factor
*QLr.DFI-7BL*	*TraesCS7B01G444000*	*TraesCS7B02G444000*	MYB transcription factor
*QLr.DFI-7DL*	*TraesCS7D01G426700*	*TraesCS7D02G426700*	Ethylene-responsive transcription factor
*QLr.DFI-7DL*	*TraesCS7D01G427100*	*TraesCS7D02G427100*	F-box family protein
*QLr.DFI-7DL*	*TraesCS7D01G427200*	*TraesCS7D02G427200*	F-box/LRR-repeat protein
*QLr.DFI-7DL*	*TraesCS7D01G427600*	*TraesCS7D02G427600*	F-box protein
*QLr.DFI-7DL*	*TraesCS7D01G429500*	*TraesCS7D02G429500*	The putative isease resistance protein (TIR-NBS-LRR class)
*QLr.DFI-7DL*	*TraesCS7D01G429600*	*TraesCS7D02G429600*	Putative disease resistance protein (TIR-NBS-LRR class)
*QLr.DFI-7DL*	*TraesCS7D01G430100*	*TraesCS7D02G430100*	F-box protein
*QLr.DFI-7DL*	*TraesCS7D01G430200*	*TraesCS7D02G430200*	F-box family protein
*QLr.DFI-7DL*	*TraesCS7D01G430600*	*TraesCS7D02G430600*	BTB/POZ domain containing protein
*QLr.DFI-7DL*	*TraesCS7D01G432500*	*TraesCS7D02G432500*	F-box domain containing protein-like
*QLr.DFI-7DL*	*TraesCS7D01G432600*	*TraesCS7D02G432600*	F-box and JmjC domain-containing protein

**Table A4:** KASP markers developed for the 5 leaf rust resistance loci identified in the Liang66-S/Hengmai28 RIL population.

KASP	QTL	Chr.	FAM	HEX	Common
*K-LR-2BL*	*QLr.DFI-2BL*	2B	GAAGGTGACCAAGTTCATGCTCACCTGTTCTTTTGCGTTTGT	GAAGGTCGGAGTCAACGGATTCACCTGTTCTTTTGCGTTTGC	CAAGATGGAGAAAAAGAAAAGGGG
*K-LR-3BS*	*QLr.DFI-3BS*	3B	GAAGGTGACCAAGTTCATGCTGTGAGATTCTTAGACATGTGGGAA	GAAGGTCGGAGTCAACGGATTGTGAGATTCTTAGACATGTGGGAG	TGAAATTTCTCCGTGAGAACAACT
*K-LR-3BL*	*QLr.DFI-3BL*	3B	GAAGGTGACCAAGTTCATGCTGATGCTTTTCGGGCTCAGA	GAAGGTCGGAGTCAACGGATTGATGCTTTTCGGGCTCAGG	GGGATCTCGGTCTCTGGAAT
*K-LR-7BL*	*QLr.DFI-7BL*	7B	GAAGGTGACCAAGTTCATGCTTACAGAGTGTAGATGCGCCA	GAAGGTCGGAGTCAACGGATTTACAGAGTGTAGATGCGCCG	AGTCCAGTTCCATCAGCTGA
*K-LR-7DL*	*QLr.DFI-7DL*	7D	GAAGGTGACCAAGTTCATGCTGCGATGAGTTTAAACGTGTCATT	GAAGGTCGGAGTCAACGGATTGCGATGAGTTTAAACGTGTCATC	AGGTCATGCAGAGAGAACAAGA

FAM and HEX are indicated by underline.

**Table A5:** The diverse panel used for validate the KASP markers *K-LR-3BS* and *K-LR-7BL*.

Number	Name	MDS for Leaf Rust	*K-LR-3BS*	*K-LR-7BL*
1	Bainong3217	37.5	AA	GG
2	Bainong5819	15	AG	AA
3	Bainong607	22.5	GG	AA
4	Bima1	85	AG	GG
5	Cunmai20	15	AA	GG
6	Fengchan3	45	GG	AA
7	Fengdecun21	52.5	AA	AA
8	Fengdecunmai20	7.5	GG	GG
9	Fengdecunmai21	17.5	GG	GG
10	Fengdecunmai24	62.5	GG	GG
11	Fengdecunmai8	22.5	AG	AA
12	Gaoyou503	80	AG	GG
13	Guan35	70	GG	GG
14	Hemai029	50	AA	-
15	Heng7228	42.5	GG	AA
16	Huaimai18	20	AG	AA
17	Huaimai20	22.5	AA	AA
18	Huaimai21	55	AA	GG
19	Huaimai46	25	GG	GG
20	Huapei5	57.5	GG	AA
21	Jimai20	7.5	GG	AA
22	Jimai44	60	AA	GG
23	Jinan13	30	AA	AG
24	Jinan17	50	AA	GG
25	Jining16	20	AG	AA
26	Jinmai61	10	GG	-
27	Jishi02-1	12.5	AG	AA
28	Lankao24	52.5	AA	GG
29	Lankao906	37.5	GG	AA
30	Liangxing619	35	GG	AA
31	Liang66-S	25	GG	AG
32	Liangxing99	35	GG	AG
33	Linhan2	67.5	AA	AA
34	Linmai4	25	GG	GG
35	Lumai15	20	AG	GG
36	Lumai23	45	AA	-
37	Lumai5	60	AG	AG
38	Lumai7	10	GG	AG
39	Lumai8	80	AA	GG
40	Lumai9	80	AG	-
41	Luohan2	40	GG	GG
42	Luomai42	32.5	GG	AA
43	Luomai45	42.5	AA	AA
44	Luomai68	25	AA	AA
45	Luyuan158	52.5	GG	GG
46	Luyuan502	70	GG	AA
47	Neixiang188	32.5	AA	AA
48	Neixiang5	72.5	AA	AA
49	Ruihua1426	42.5	GG	AA
50	Shaan354	10	GG	AA
51	Shaanmai509	30	GG	AA
52	Shaanyou225	12.5	AG	GG
53	Shannong05-066	17.5	AA	AA
54	Shannong17	77.5	GG	AA
55	Shannong30	82.5	AA	GG
56	Shannong38	32.5	AA	AA
57	Shannong48	12.5	AA	AA
58	Shi4185	55	AA	GG
59	Shijiazhuang8	50	AA	GG
60	Shiluan02-1	15	AA	AA
61	Su0663	25	AG	AA
62	Taikemai33	22.5	GG	GG
63	Taishan1	35	GG	AA
64	Wan23094	40	GG	AA
65	Wanmai29	20	GG	AG
66	Wanmai33	12.5	GG	AG
67	Wanmai38	25	GG	GG
68	Wanmai50	50	AA	AA
69	Wanmai53	50	AA	AA
70	Wennong14	55	GG	AA
71	Wunong148	5.5	GG	GG
72	Xiaoyan54	35	AA	AG
73	Xiaoyan6	47.5	GG	-
74	Xiaoyan81	17.5	GG	AA
75	Xinmai32	45	AA	AA
76	Xinmai45	37.5	AA	AG
77	Xinmai9408	5	AA	AG
78	Xinong1376	62.5	GG	AA
79	Xinong291	42.5	AA	AA
80	Xinong979-005	22.5	GG	AA
81	Xumai33	32.5	GG	AG
82	Xumai35	40	AA	AG
83	Xumai45	50	GG	GG
84	Yan1212	10	AA	GG
85	Yumai2	50	AG	AG
86	Yumai35	65	GG	GG
87	Yumai57	57.5	AA	GG
88	Yumai7	60	AA	GG
89	Zheng7698	25	GG	GG
90	Zhengmai136	55	GG	GG
91	Zhengmai1860	20	AA	AA
92	Zhengmai366	27.5	AA	GG
93	Zhengmai369	22.5	GG	GG
94	ZhengYin1	32.5	AA	AG
95	Zhong892	55	AA	GG
96	Zhongke166	30	GG	GG
97	Zhongmai173	60	AA	AG
98	Zhongmai23	15	AA	AA
99	Zhongxinmai998	82.5	AA	GG
100	Zhongyu9	25	GG	-
101	Zhou8425B	17.5	GG	AG
102	Zhoumai11	30	AA	AA
103	Zhoumai12	22.5	AA	AA
104	Zhoumai16	50	AA	AA
105	Zhoumai18	37.5	GG	-
106	Zhoumai26	42.5	AG	AA
107	Zhoumai27	40	GG	AA
108	Zhoumai28	10	GG	AA
109	Zhoumai30	22.5	GG	GG
110	Zhoumai31	57.5	GG	AA
111	Zhoumai33	10	GG	AA
112	Zhoumai36	12.5	AA	GG
113	Zhoumai38	7.5	GG	AA
114	Zhoumai40	30	GG	GG
115	Zhoumai49	20	GG	GG
116	Zhouyuan9396	20	GG	GG
117	Zhumai762	10	GG	AA
118	Zimai12	50	GG	GG
119	Zimai19	45	GG	GG
120	Zixuan2	82.5	AA	GG

**Table A6:** Summary of previously reported leaf rust resistance genes/QTL on chromosomes 2B, 3B, 7B, and 7D.

Chr.	Gene/QTL	Linked Markers	Physical Position (IWGSC v1.0) (Mb)	Reference
2B	*Lr23*	*Xgwm148*, *Xbarc55*, *Xwmc453*	8.5–9.0 Mb	[50,51]
2B	*Lr13*	*Xwmc764*, *Xbarc55*, *Xrwgs119*, *Xcfd51*	9.35–9.47 Mb	[50,52]
2B	*QLr.cimm-2B*	*Xgwm148*, *Xgwm374*	~8.0–20.0 Mb	[53]
2B	*Lr16*	*Xwmc764*, *Xbarc55*, *Xgwm210*	~14.5–15.5 Mb	[54]
2B	*Lr48*	*Xgwm429*, *Xbarc55*, *Xwmc257*	31.0–33.0 Mb	[55]
2B	*QLr.hebau-2B.1*	*Xwmc770*, *Xbarc91*	~30.0–35.0 Mb	[56]
2B	*QLr.DFI-2BL*	*Bob-White_c2512_215–Kukri_c13624_201*	689.9–692.5 Mb	[25,26]; This study
2B	*Lr58*	*Xgwm388*, *Xbarc91*	775–780 Mb	[57]
2B	*Lr35*	*Xgwm210*, *Xgwm319*	Alien translocation	[58]
3B	*QLr.DFI-3BS*	*Kukri_rep_c94244_223*–*Tdurum_contig49804_392*	2.3–4.1 Mb	[26,27]; This study
3B	*QLr.DFI-3BL*	*Ra_c33692_1499*–*Tdurum_contig12800_1004*	562.4–567.2 Mb	[28]; This study
3B	*QLr.cimm-3B.1*	*Xwmc533*, *Xbarc133*	665–670 Mb	[53]
3B	*Lr27*	*Xgwm533*, *csSr2*, *Xwmc505*	~668.3 Mb	[50,59]
3B	*QLr.ksu-3B.1*	*Xbarc164*, *Xwmc505*, *Xgwm493*	670–690 Mb	[60]
3B	*Lr49*	*Xgwm247*, *Xwmc231*, *Xbarc77*	730–760 Mb	[61]
3B	*QLr.csiro-3B*	*Xgwm340*, *Xgwm299*	730–750 Mb	[62]
3B	*QLr.sfrs-3B*	*Xgwm247*, *Xbarc77*	730–760 Mb	[63]
7B	*Lr68*	*Xgwm146*, *csSNP856*, *Xbarc32*	703–706 Mb	[9,29,64]
7B	*QLr.cimm-7B*	*Xwmc526*, *Xgwm146*	~703–706 Mb	[53]
7B	*QLr.DFI-7BL*	*BS00055584_51*–*BS00022636_51*	704.6–708.1 Mb	[9,29,30,31,64]; This study
7B	*QLr.csiro-7B*	*Xgwm344*, *Xwmc526*	~710–730 Mb	[62]
7B	*Lr14a*	*Xwmc526*, *Xgwm146*, *Xbarc32*	729–732 Mb	[65]
7B	*Lr14b*	*Xwmc526*, *Xgwm146*	729–732 Mb	[50]
7D	*Lr29*	*Xwmc488*, *Xbarc70*	63–71 Mb	[66]
7D	*QLr.cimm-7D*	*wPt-7290*, *Xbarc70*	~65–72 Mb	[53]
7D	*QLr.DFI-7DL*	*D_contig09547_691*–*RAC875_c18043_369*	547.3–550.1 Mb	[32]; This study
7D	*Lr19*	*Sdpe*, *XsdauK279*, *Xwg380*	623–638 Mb	[32,67]
7D	*Lr34*	*csLV34*, *Xgwm295*, *Xwmc525*, *Lr34Exon-SNP*	655.4–657.1 Mb	[68]
7D	*QLr.sfrs-7D*	*Xgwm295*, *Xwmc525*	654–658 Mb	[63]
7D	*Lr38*	*Xgwm121*, *Xgdm88*	Alien translocation on 7DS	[69]
