# Correction: Bo et al. QTL Mapping for Leaf Rust Resistance in a Wheat Recombinant Inbred Line Population of Liang66-S/Hengmai28. *Plants* 2026, *15*, 2684

**DOI:** 10.3390/plants15203232

**Published:** 2026-10-21

**Authors:** Cunyao Bo, Chenyang Pang, Xianghai Meng, Ding Li, Minghui Zhao, Chuncai Shen, Qiang Li, Wenchen Qiao

**Affiliations:** 1 National Key Laboratory of Wheat Improvement, College of Agronomy, Shandong Agricultural University, Tai’an 271018, China; bocunyao1989@126.com (C.B.); pangcy118@foxmail.com (C.P.); 2 Dryland Farming Institute, Hebei Academy of Agriculture and Forestry Sciences, Hengshui 053000, China; mengxianghai5229@163.com (X.M.); nxld854@163.com (D.L.); mnzhaominghui@163.com (M.Z.); shencc_0419@126.com (C.S.); 3 Hebei Provincial Key Laboratory of Crops Drought Resistance Research, Hengshui 053000, China

In the original publication [1], references [21], [22], [35], [56], [60] and [63] were incorrectly cited. They have now been corrected, with the reference order unchanged.

21. Zhao, M.; Ge, Y.; Xu, Z.; Ouyang, X.; Jia, Y.; Liu, J.; Zhang, M.; An, Y. A BTB/POZ domain-containing protein negatively regulates plant immunity in *Nicotiana benthamiana*. *Biochem. Biophys. Res. Commun.* **2022**, *600*, 54–59.
22. Zhao, K.; Tian, H.; Zhou, L.; Liu, C.; Wang, H.; Yang, D.; Guan, J.; Zhang, S.; Huang, X. Genome-wide identification of calcium-dependent protein kinase (CDPK) family genes in pear (Pyrus bretschneideri) and functional characterization of PbrCDPK8 in black spot tolerance. *Hortic. Plant J.* 2025, *in press*. https://doi.org/10.1016/j.hpj.2025.07.025.
35. Gou, M.; Shi, Z.; Zhu, Y.; Bao, Z.; Wang, G.; Hua, J. The F-box protein CPR1/CPR30 negatively regulates R protein SNC1 accumulation. *Plant J.* **2012**, *69*, 411–420.
56. Zhou, Y.; Gao, P.; Miao, X.; Gebrewahid, T.W.; Jiao, Y.; Zhang, P.P.; Cao, L.Q.; Zhang, X.L.; Li, Z.F. QTL mapping of adult-plant resistance to leaf and stripe rust in wheat cross L224-3/Zhengzhou5389. *Euphytica* **2023**, *219*, 105.
60. Hou, W.; Lu, Q.; Ma, L.; Sun, X.; Wang, L.; Nie, J.; Guo, P.; Liu, T.; Li, Z.; Sun, C.; et al. Mapping of quantitative trait loci for leaf rust resistance in the wheat population ‘Xinmai 26/Zhoumai 22’. *J. Exp. Bot.* **2023**, *74*, 3019–3032.
63. Landjeva, S.; Neumann, K.; Lohwasser, U.; Börner, A. Molecular mapping of genomic regions associated with wheat seedling growth under osmotic stress. *Biol. Plant.* **2008**, *52*, 259–266.

The authors state that the scientific conclusions are unaffected. This correction was approved by the Academic Editor. The original publication has also been updated.
